# Barriers to Development of an App for Improving Staff-Family Communication at Israeli Geriatric Facilities

**DOI:** 10.1093/geroni/igaa057.623

**Published:** 2020-12-16

**Authors:** Rinat Cohen, Gal Maydan, Shai Brill, Jiska Cohen-Mansfield

**Affiliations:** 1 School of Public Health, Sackler Faculty of Medicine, Tel Aviv University, Hadera, Israel; 2 Beit Rivka Medical Center, Petach Tikva, Israel; 3 Clalit Health Services, Tel Aviv, Israel; 4 Tel Aviv University, Tel Aviv, Israel

## Abstract

Family caregivers (FCs) of institutionalized noncommunicative older persons reported multiple unmet communication needs focusing on the need to receive reliable and regular updates on the patient’s condition. We have developed a mobile app for improving communication between FCs and healthcare professionals (HPs), based on 152 interviews with FCs and 13 discussion groups with HPs from four Israeli geriatric facilities. Both parties participated in app planning, tailoring it to their needs and abilities. App use implementation encountered major obstacles including the bureaucratic process concerning signing contracts between the university and software development firms, which hindered the process for a full year; data security department required disproportionate security levels that interfered with user experience and delayed the development process; the study’s definition varied across different ethics/Helsinki committees (Institutional Review Boards; IRBs), which led to different demands, e.g., insurance for medical clinical trials although no drugs or medical device were involved; lack of cooperation by mid-level staff members despite the institutional adoption of the app project; low utilization by HPs resulted in FCs not receiving timely responses. Despite these and other obstacles, we tested app use for 15 months in one facility in a pre-post-design with intervention and control groups, and we have since begun testing it in another facility. FCs who had used the app had positive feedback and wished to continue using it. App use optimization requires implementation planning, assimilating changes in each facility’s work procedures and HP’s engagement and motivation and thus depends on institutional procedures and politics.

